# Incidence of edentulism among older adults using the Korean National Health Insurance Service database, 2013-2018

**DOI:** 10.4178/epih.e2022091

**Published:** 2022-10-17

**Authors:** Hyeonjeong Go, Eun-Kyong Kim, Hoi-In Jung, Song Vogue Ahn, Hosung Shin, Atsuo Amano, Youn-Hee Choi

**Affiliations:** 1 Department of Preventive Dentistry, School of Dentistry, Kyungpook National University, Daegu, Korea; 2 Department of Dental Hygiene, Kyungpook National University, Sangju, Korea; 3 Department of Preventive Dentistry and Public Oral Health, Yonsei University College of Dentistry, Seoul, Korea; 4 Department of Health Convergence, Ewha Womans University, Seoul, Korea; 5 Department of Social and Humanity in Dentistry, Wonkwang University School of Dentistry, Iksan, Korea; 6 Department of Preventive Dentistry, Osaka University Graduate School of Dentistry, Osaka, Japan; 7 Institute for Translational Research in Dentistry, Kyungpook National University, Daegu, Korea

**Keywords:** Edentulism, Incidence, Korean National Health Service Insurance, Cohort database, Elderly, Tooth loss

## Abstract

**OBJECTIVES:**

Population aging is rapidly accelerating worldwide. Oral diseases related to aging are also on the rise. This study examined trends in the incidence of edentulism among the older Korean population using data from the Korean National Health Insurance Service (KNHIS).

**METHODS:**

Data on older adults, aged ≥75 years of age, were obtained from the KNHIS for the period 2013-2018. Edentulism was defined as a treatment history of complete dentures in the KNHIS database. The exclusion criteria consisted of both disease codes and treatment codes related to conservative dental treatment, including periodontal and extraction treatment afterward. Crude incidence rates (CIRs) and age-standardized incidence rates (AIRs) with 95% confidence intervals were calculated and reported per 100,000 person-years by the direct method. Trends were tested by Cochrane Armitage models.

**RESULTS:**

Statistically significant increasing trends in both CIRs and AIRs were found among the older Korean population registered in the KNHIS (CIRs, 707.92 to 895.92; AIRs, 705.11 to 889.68; p<0.01). The incidence tended to increase in both genders (p<0.01). Both CIRs and AIRs in specific regions also showed slight but significant annual increases except for Jeju Island (p<0.01 or <0.05). The incidence showed increasing trends (p<0.01) in all income quintiles apart from the highest quintile. The edentulism incidence was highest in the lowest income group (the first quintile).

**CONCLUSIONS:**

Our data showed that the incidence of edentulism among the elderly showed an increasing trend from 2013 to 2018. This result provides a basis for future epidemiological studies on the incidence of edentulism in the older Korean population.

## GRAPHICAL ABSTRACT

[Fig f2-epih-44-e2022091]

## INTRODUCTION

The worldwide aging population is rapidly accelerating. Globally, Korea is one of the world’s fastest aging countries, and fast becoming a “hyper-aging society,” in which the proportion of older adults aged 65 years and over exceeds 20%. Oral diseases related to aging are also on the rise. This is a cause for concern, as poor oral health generally lowers the quality of life. Edentulism is the state of complete tooth loss (i.e., not having a single natural tooth). Tooth loss can be explained not only as the cumulative effect of oral diseases, especially dental caries and periodontitis, but also as a result of treatment decisions [1,2] and the value placed on natural teeth. As such, both biological and social factors are involved in edentulism [3,4]. Edentulism leads to the deterioration of basic functions such as mastication, pronunciation, and facial appearance. Consequently, these changes can lead to insufficient food intake and poor nutrition [5-7], weight loss [8,9], increased risks of systemic diseases, and poor quality of life [10-13]. According to previous studies, when considering the number of years of loss due to poor health, disability or early death measured by disability-adjusted life years, severe tooth loss is considered a major disease burden for those aged 60 years or more [14-16]. According to the fourth Korea National Health and Nutrition Examination Survey (KNHANES) (2007-2009) conducted by the Korea Centers for Disease Control and Prevention (now known as the Korea Disease Control and Prevention Agency), the mean number of teeth in those aged 65-69 years and 75-79 years was 19.4 and 13.6, respectively [17]. Edentulism is a public health burden for older people and clearly affects primary care practices [18]. In Korea, removable complete and partial dentures, and dental implant services for the elderly population (aged ≥ 65) are provided by the Korean National Health Insurance Service (KNHIS). However, the oral disease burden among older individuals remains present due to gaps in health insurance coverage, which are frequently underestimated.

Previous surveys on trends in the prevalence of edentulism using national data have been conducted in different countries. For example, Slade et al. [19] assessed the trend of edentulism using data from national health surveys in the United States, and Cardoso et al. [20] reported the prevalence of edentulism from Brazil’s National Oral Health Survey. In Korea, Yu et al. [21] also reported the prevalence of edentulism among Korean adults using raw data from the KNHANES. However, studies on the incidence of edentulism are rare, and available research studies are mainly cross-sectional in design or time-series. These studies have limited sample sizes; thus, significant epidemiological relationships may not have been recognized. Longitudinal studies with large-scale sample sizes are warranted.

The purpose of this study was to further understand this process, guide policy decision-making, and provide support for expanding the coverage of health insurance. Thus, this study evaluated the incidence of edentulism in the 75-year and older population registered in the KNHIS. The results may be used as a reference for developing countermeasures and new polices.

## MATERIALS AND METHODS

### Study design and data source

We conducted a retrospective population-based cohort study using the KNHIS-customized database from 2013 to 2018, which was the total period of the available dataset. The KNHIS database included records for the entire Korean population. It is a single-payer program that has covered the entire Korean population of approximately 50 million since 2002, through either the National Health Insurance System (97%) or Medical Aid (3%) [22,23]. The KNHIS-customized database contained records of dental and medical treatment information requested by researchers and collected by the National Health Insurance Corporation. The database is based on the date of the claim for the use of hospitals, clinics, and pharmacies, including national health insurance qualification data containing medical statements, disease codes, treatment codes, and prescription details, from 2002 to 2018. The disease codes in KNHIS were derived from the Korean Standard Classification of Disease, 7th revision, which was modified from the International Classification of Disease, 10th revision. The treatment classification according to Korean standard drug classification codes was also included.

### Study population and the operational definition of edentulism

**Figure 1.**
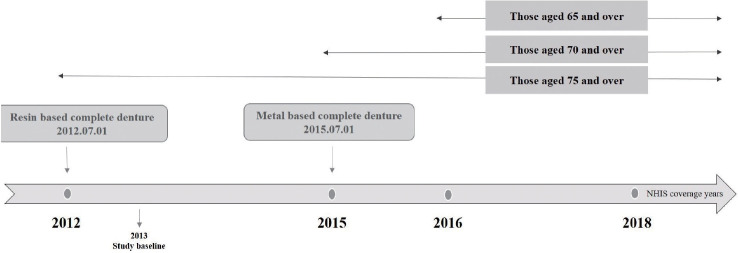
Description of the Korean National Health Insurance Service coverage of complete dentures in Korea. NHIS, National Health Insurance Service.

In this study, research was conducted using the total data, which included the elderly aged ≥ 75 from 2013 to 2018, when complete dentures were covered by insurance. To analyze trends in the incidence of complete edentulism in older adults, those with a history of complete dentures diagnosis and treatment from January 1, 2013 to December 31, 2018, were collected from the KNHIS-customized cohort data. The incidence of complete edentulism was calculated for older adults, aged 75 years or older, who were included within the study period and were covered by insurance benefits. The age for insurance benefits has expanded to those who are ≥ 75 years old from July 1, 2012, the population aged ≥ 70 years from July 1, 2015, and the over-65 population from July 1, 2016 (Figure 1). To compare the trend of incidence for each year on equal terms within a specified study period, older adults aged ≥ 75 years who were the subjects of insurance coverage for complete dentures throughout the entire study period, not those who aged ≥ 65, were selected.

As shown in Table 1, those with a K08.1 disease code as the diagnosis who received treatment corresponding to the included treatment codes, from January 1, 2013, to December 31, 2018, were regarded as edentulous patients. Those who were subsequently diagnosed with K02, K04, K05, or K08.3, which are related to teeth, and had treatment codes corresponding to the exclusion criteria, after complete denture treatment, were considered to have teeth and were excluded, as described in a previous study [24]. Furthermore, the deceased and those with missing data for relevant parameters were excluded.

### Statistical analysis

We analyzed the frequency of receiving treatment of UA101-109 and UA501-509, corresponding to the first stage of complete denture treatment, from 2013 to 2018 among those with a K08.1 diagnosis. Of note, the insurance coverage period for complete dentures is once every 7 years [25]. Thus, cases that satisfied the diagnostic criteria of edentulism first occurred during the study period from 2013 to 2018. Therefore, we defined receiving a K08.1 diagnosis and who received treatment with a code of UA101-109 or UA501-509 as the first time point of edentulism. Using this definition, the incidence of edentulism among older Korean adults aged ≥ 75 years was investigated.

Based on the KNHIS-customized database, the incidence of edentulism per 100,000 people was calculated for each year. The incidence of edentulism by the total population, gender, regions, and income among older adults was also calculated. The incidence was calculated by dividing the number of edentulous patients with complete dentures by the total population of Koreans aged ≥ 75 years registered in the KNHIS annually as of the end of the year from the KNHIS Statistical Yearbook, and multiplying by 100,000.

Regions were divided into 10 categories: Seoul; Busan; Ulsan and South Gyeongsang Province ; Daegu and North Gyeongsang Province; Incheon and Gyeonggi Province; Gwangju and South Jeolla Province; Gangwon Province; North Chungcheong Province; South Chungcheong Province; Daejeon, North Jeolla Province, and Sejong ; and Jeju Special Self-Governing Province.

Income was divided into 5 quintiles. The lowest income segment was the first quintile (the lowest 20%). The highest income quintile was defined as the top 20%. Health insurance eligibility standards (head of household, family member, and Medical Aid beneficiaries) were also considered.

All statistical analyses were performed using SAS version 9.4 (SAS Institute Inc., Cary, NC, USA). The chi-square test was used to analyze descriptive statistics. Frequency data were presented as the number of cases (n) with percentage (%). Age-standardized incidence rates (AIRs) with 95% confidence intervals (CIs) were calculated and reported 100,000 person-years by the direct method [26]. Statistical significance in differences between crude incidence rates (CIRs) and AIRs was determined based on whether CIRs were included within the 95% CI of AIRs. The incidence trends were evaluated by the Cochran Armitage trend test.

### Ethics statement

Institutional Review Board/Ethics Committee approval was obtained from the Wonkwang University Institutional Review Board (WKIRB-201911-SB-082), the Kyungpook National University Institutional Review Board (KNU-2021-0489), and the National Evidence-based Healthcare Collaborating Agency (NECAIRB21-009).

## RESULTS

Table 2 shows the population of Koreans aged ≥ 75 insured through the KNHIS and trends in the incidence of edentulism. The number of older adults showed an average overall increase of 5.38% over the 6 years from 2013 to 2018. Both the CIRs and the AIRs of edentulism showed a statistically significant increasing trend (CIRs: 707.92 to 895.92; AIRs: 705.11 to 889.68; p< 0.01). The incidence slightly decreased in 2017 and increased in 2018. The average rate of change in the CIR of edentulism was 3.18% over the 6 years from 2013 to 2018, and that of the AIR was 3.06%. Between 2016 and 2017, the CIR decreased by 5.83% from 751.73 to 707.92 per 100,000 people, and then increased by 26.56% in 2018; the AIR decreased by 5.78% between 2016 and 2017, and then increased by 26.18% in 2018. The incidence gradually increased annually in both genders (p< 0.01), with a slightly higher incidence of edentulism in men than women. The annual average increase in incidence from 2013 to 2018 was 3.68% in men and 2.80% in women.

As shown in Table 3, both the CIRs and the AIRs significantly increased annually in each region except for Jeju Island (p< 0.01 or < 0.05). The average difference between the CIRs and AIRs was 1.16-fold, (125.23 per 100,000 persons) in Gwangju and South Jeolla Province, approximately 0.84-fold (-140.00 per 100,000 persons) in North Jeolla Province, and 0.94-fold (-65.07 per 100,000 persons) in Daegu and North Gyeongsang Province. In particular, Daegu and North Gyeongsang Province, and North Chungcheong Province showed higher edentulism incidence rates than other regions. In 2017, the edentulism incidence by region was generally lower than in the other years. The highest annual average rate of change in any region from 2013 to 2018 was a 9.55% increase in Daejeon, Chungcheongnam Province, and Sejong, while the rest of the regions showed increases of approximately 4%.

The incidence of edentulism showed an increasing trend between 2013 and 2018 in all quintiles except for the highest-income group (p< 0.01). The edentulism incidence was highest in the first quintile (the lowest income group) (Table 4). The annual average rate of change in edentulism incidence by income group from 2013 to 2018 was an increase of 2.65%.

## DISCUSSION

This study examined the incidence of edentulism in the elderly Korean population (aged ≥ 75 years) from 2013 to 2018 using data from the KNHIS. Complete dentures were first covered by the KNHIS in July 2012. The incidence of edentulism among older adults gradually increased between 2013 and 2018. The edentulism incidence also differed by region. Furthermore, higher incidence was observed in lower-income quintiles.

In a recent study, edentulism in older adults (aged ≥ 80 years) showed an increasing trend from 2007 to 2017 [21]. Korea is moving toward an aging society with an increasingly older population, and the edentulism incidence among older Koreans in this study also slightly increased. According to Statistics Korea, the medical expenses per person aged ≥ 65 years continuously increase slightly every year [27]. The reason for the increase in medical expenses for the elderly is the increased prevalence of chronic diseases. Moreover, 70.9% of people with chronic diseases have 3 or more comorbid chronic diseases [28]. Many studies have shown that chronic systematic diseases and chronic periodontitis can cause tooth loss [29-33]. Thus, an increase in the number of older people with high levels of morbidity related to chronic diseases and the complex interactions between oral diseases and chronic diseases probably contributed to the increasing incidence trend of edentulism. Moreover, Silva-Junior et al. [1] reported a higher incidence of tooth loss in older adults within 4 years. Overall, the incidence of edentulism in older adults has shown a slightly increasing trend. As a result, greater efforts and different strategies are needed to achieve the “oral health in older adults” goal of Health Plan 2030.

Our results showed that there were more women than men in the older population. Conversely, men had a slightly higher incidence of edentulism than women. A recent study in Japan also found that men, who experienced more risk factors such as smoking, had a higher risk of tooth loss than women [34]. The authors observed that the incidence of edentulism among older adults was affected by residential region and income. Previous studies [35-37] have also reported that the number of people with tooth loss was associated with socioeconomic level. In this study, differences in the incidence between urban and rural regions were not analyzed because we did not perform the analysis according to the county (*gun*) level. However, we confirmed that the incidence of edentulism differed by units of cities and provinces. The edentulism incidence was the highest in Daegu and North Gyeongsang Province. This observation suggests that there is a need to develop better preventive oral health programs to improve the oral health of older adults living in that region. The annual average rate of change in the incidence of edentulism by region from 2013 to 2018 was the highest in Daejeon, South Chungcheong Province, and Sejong, at approximately 10%. Looking at income, our results showed that the edentulism incidence was the highest in the first quintile (lowest income group). According to a report presenting data from each region in Korea in 2017, the relative poverty of older adults in the bottom 20% of income rose somewhat in Daejeon and South Chungcheong Province [38]. This is speculated to have had some effects on the incidence of edentulism in that region, since edentulism is affected by financial conditions. To address this issue, government-led innovations in oral health policies, including effective oral health promotion among those with lower household incomes, are warranted.

The strengths of this study include the use of KNHIS data, as a representative population-based cohort dataset. The database used for analyses was large, extensive, and stable because it was constructed based on national health insurance data generated by the government or public institutions [22]. Therefore, these results can be used by policy-makers to create higher value-adding policies. Our results may also provide fundamental data for improving the oral health quality of life of older adults and ensure timely responses to a rapidly changing and aging society.

This study has several limitations. First, the operational definition of edentulism used in this study was based on health insurance records, not medical records. Since edentulism was not defined based on direct diagnoses from medical records and examinations, the results may have been underestimated. Therefore, further studies should be conducted to increase the sensitivity and specificity of the edentulism diagnosis for determining edentulism incidence. Second, we did not include data on treatments not covered by insurance. However, it became less common for older adults who paid out-of-pocket for uninsured dental treatment after the health insurance coverage of complete dentures [39]. Furthermore, invasive treatments such as dental implants or overdentures may be relatively limited in the older population (aged ≥ 75 years) because of their general health condition. Thus, the population in our study might have been less affected by these factors. Finally, since our analyses were based on data from the KNHIS, the increased incidence of edentulism may have been affected by the policy changes in insurance coverage that relate to oral health diseases and edentulism.

This data shows that the incidence of edentulism among older Korean adults, aged ≥ 75 years, registered in the KNHIS statistically significantly increased from 2013 to 2018 (i.e., since 2012, when complete dentures were first covered by the NHIS). Men had a slightly higher incidence of edentulism than women. Moreover, the incidence differed by region and income. Importantly, people in lower income quintiles had higher edentulism incidence. This result provides a basis for future epidemiological studies on the incidence of edentulism in the older population in Korea.

## Figures and Tables

**Figure f2-epih-44-e2022091:**
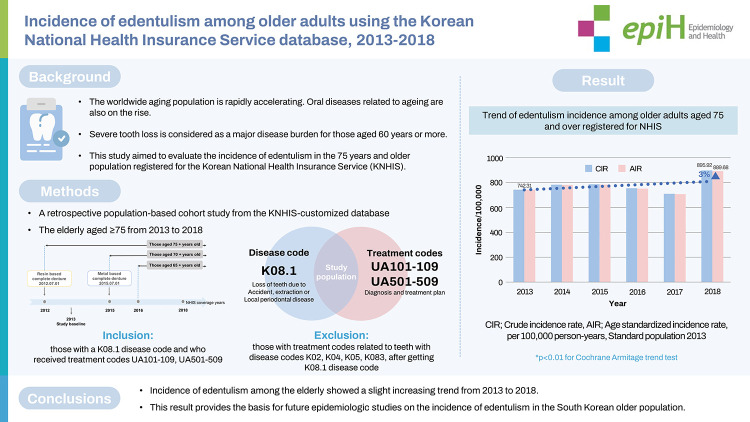


**Table 1.** Inclusion and exclusion criteria by Korean Standard Classifica tion of Diseases, version 7 (KCD-7) codes1 for edentulism

Codes			Criteria
Inclusion			
	Disease code^1^		
		K08.1	Loss of teeth due to accident, extraction or local periodontal disease
	Treatment codes^2^		
		UA101-109	Diagnosis and treatment plan for resin based complete denture
		UA501-509	Diagnosis and treatment plan for metal based complete denture
Exclusion			
	Treatment codes^2^		
		U0002	Reamer of file
		U0010	Simple treatment
		U0011	Dental sedative filing
		U0020	Pulp capping
		U0041	Desensitizing treatment-topical application, iontophoresis
		U0060	Access cavity preparation
		U0074	Treatment for one visit filing
		U0090	Pulpotomy
		U0101	Pulp extirpation
		U0111	Root canal irrigation
		U0116	Root canal enlargement
		U0119	Root canal shaping
		U0121	Root canal filling with single cone method
		U0126	Root canal filling with condensation method
		U0131	Amalgam filling (1 surface)
		U0132	Amalgam filling (2 surfaces)
		U0133	Amalgam filling (3 surfaces)
		U0134	Amalgam filling (≥4 surfaces)
		U0135	Composite resin filling (1 surface)
		U0136	Composite resin filling (2 surfaces)
		U0137	Composite resin filling (3 surfaces)
		U0138	Composite resin filling (≥4 surfaces)
		U0140	Rubber dam application
		U0151	Cavity preparation (1 surface)
		U0152	Cavity preparation (2 surfaces)
		U0153	Cavity preparation (3 surfaces)
		U0154	Cavity preparation (≥4 surfaces)
		U0220	Restoration polishing
		U0210	Emergency pulp treatment
		U0220	Recementation
		U0239	Light curing composite resin restoration (1 surface)
		U0240	Light curing composite resin restoration (2 surfaces)
		U0241	Light curing composite resin restoration (≥3 surfaces)
		UX001	Desensitizing treatment-laser treatment, dentin adhesive application
		U2232	Scaling
		U2233	Scaling before periodontal treatment
		U4412	Extraction (anterior tooth)
		U4413	Extraction (posterior tooth)
		U4414	Extraction (complicated extraction)

1 The disease classification according to the KCD-7 was used.

2 The treatment classification according to the Korean standardized drug classification codes was used; After receiving a K08.1 disease code, patients with treatment codes for the disease codes of K02, KO4, KO5, KO83 (related to teeth) were considered to have teeth and excluded.

**Table 2.** Trends in edentulism incidence in older adults aged 75 and over registered in the KNHIS

Year	Incidence rate (per 100,000)^**^				Incidence case (n)			The no. of population^1^		
	CIR	AIR (95% CI)	Men	Women	Total	Men	Women	Total	Men	Women
2013	742.31	1.00 (reference)	771.35	726.84	17,981	6,494	11,487	2,422,300	841,904	1,580,396
2014	779.86	778.20 (731.46, 753.16)	773.13	783.52	20,262	7,079	13,183	2,598,158	915,633	1,682,525
2015	784.41	781.38 (767.09 , 789.31)	802.08	774.62	21,576	7,861	13,715	2,750,617	980,075	1,770,542
2016	751.73	748.40 (770.25, 792.51)	779.60	735.99	22,031	8,248	13,783	2,930,709	1,057,981	1,872,728
2017	707.92	705.11 (737.50, 759.29)	736.23	691.50	22,448	8,569	13,879	3,170,983	1,163,908	2,07,75
2018	895.92	889.68 (877.80, 901.56)	958.27	859.29	29,724	11,765	17,959	3,317,714	1,227,729	2,089,985

KNHIS, Korean National Health Insurance Service; CIR, crude incidence rate; AIR, age-standardized incidence rate per 100,000 person-years; standard population 2013; CI, confidence interval.

1 The number of population as of the end of the year from the National Health Insurance Statistical Yearbook.

** p<0.01 for the Cochrane Armitage trend test.

**Table 3.** Trend of edentulism incidence in older adults by residence

	2013		2014		2015		2016		2017		2018	
	CIR	AIR (95% CI)	CIR	AIR (95% CI)	CIR	AIR (95% CI)	CIR	AIR (95% CI)	CIR	AIR (95% CI)	CIR	AIR (95% CI)
Seoul^**^	482.18	1.00 (reference)	545.23	550.42 (526.86, 573.98)	528.39	525.76 (502.74, 548.79)	553.34	546.37 (522.90, 569.85)	498.70	492.27 (469.99, 514.55)	651.02	637.07 (611.72, 662.42)
Busan, Ulsan, South Gyeongsang Province^**^	955.27	-	955.53	989.16 (956.28, 1022.05)	966.57	985.21 (952.39, 1018.03)	915.00	923.53 (891.75, 955.30)	881.09	890.04 (858.85, 921.23)	1,068.75	1,060.07 (1,026.03, 1,094.12)
Daegu, North Gyeongsang Province^*^	994.61	-	1,006.77	1,110.20 (1,078.78, 1,141.61)	1,035.31	1,094.77 (1,063.57, 1,125.97)	947.68	979.99 (950.47, 1,009.51)	898.65	914.97 (886.45, 943.49)	1,131.75	1,153.91 (1,121.87, 1,185.94)
Incheon-Gyeonggi Province^**^	584.79	-	619.39	659.85 (637.79, 681.91)	630.24	647.80 (625.94, 669.66)	601.30	612.04 (590.80, 633.29)	580.64	586.12 (565.32, 606.91)	735.88	747.40 (723.92, 770.88)
Gwangju, South Jeolla Province^*^	808.71	-	897.91	795.60 (757.67, 833.53)	770.85	773.63 (736.22, 811.03)	868.01	747.65 (710.88, 784.42)	789.91	682.80 (647.66, 717.94)	1,016.64	868.43 (828.80, 908.06)
North Jeolla Province^**^	619.17	-	716.61	875.83 (825.25, 926.40)	817.05	815.73 (766.91, 864.54)	689.64	831.43 (782.15, 880.71)	649.96	770.70 (723.25, 818.14)	824.96	963.23 (910.18, 1,016.27)
Gangwon Province^**^	770.52	-	897.53	941.87 (879.84, 1003.90)	895.85	926.65 (865.12, 988.18)	897.44	919.25 (857.92, 980.53)	799.91	812.08 (754.48, 869.68)	966.36	970.15 (907.19, 1,033.10)
North Chungcheong Province^**^	950.10	-	905.18	964.66 (899.59, 1029.72)	1020.13	1,043.78 (976.10, 1111.46)	876.81	905.89 (842.84, 968.93)	898.18	925.21 (861.50, 988.93)	1,148.79	1,148.25 (1,077.27, 1,219.24)
Daejeon, South Chungcheong Province, Sejong^**^	578.40	-	846.07	814.86 (775.45, 854.26)	832.64	796.87 (757.91, 835.84)	796.05	742.78 (705.16, 780.40)	736.07	726.18 (688.98, 763.37)	999.57	977.94 (934.77, 1,021.10)
Jeju Island	724.38	-	750.99	775.55 (678.62, 872.48)	814.92	832.95 (732.49, 933.40)	723.72	727.07 (633.22, 820.92)	662.53	657.52 (568.27, 746.77)	800.78	774.54 (677.67, 871.40)

CIR, crude incidence rate; AIR, age-standardized incidence rate per 100,000 person-years; standard population 2013; CI, confidence interval.

* p<0.05,

** p<0.01 for the Cochrane-Armitage trend test.

**Table 4.** Trends in edentulism incidence among older adults by income distribution^1^

Year	First quintile^**^	Second quintile^**^	Third quintile^**^	Fourth quintile^**^	Fifth quintile
2013	829.81	777.82	798.99	759.97	743.05
2014	865.07	853.66	811.88	780.18	761.43
2015	794.33	864.78	828.31	809.83	772.93
2016	822.63	808.00	780.12	760.87	704.71
2017	744.43	747.76	737.61	715.01	671.99
2018	962.51	931.38	955.99	904.23	824.25

Rates are given per 100,000 person-years.

1 The lowest income segment (the bottom 20% of income) was the first quintile, and the highest quintile corresponded to the top 20% of income.

** p<0.01 for the Cochrane-Armitage trend test.

## References

[1] Silva-Junior MF, Batista MJ, de Sousa MD (2017). Incidence of tooth loss in adults: a 4-year population-based prospective cohort study. Int J Dent.

[2] Jafarian M, Etebarian A (2013). Reasons for extraction of permanent teeth in general dental practices in Tehran, Iran. Med Princ Pract.

[3] De Marchi RJ, Hilgert JB, Hugo FN, Santos CM, Martins AB, Padilha DM (2012). Four-year incidence and predictors of tooth loss among older adults in a southern Brazilian city. Community Dent Oral Epidemiol.

[4] Russell SL, Gordon S, Lukacs JR, Kaste LM (2013). Sex/gender differences in tooth loss and edentulism: historical perspectives, biological factors, and sociologic reasons. Dent Clin North Am.

[5] Jun MJ, Ryu SY (2016). Oral health and behavior by tooth loss: the sixth Korea National Health and Nutrition Examination Survey. J Korea Entertain Ind Assoc.

[6] Wu LL, Cheung KY, Lam PY, Gao XL (2018). Oral health indicators for risk of malnutrition in elders. J Nutr Health Aging.

[7] Lee JH, Choi JK, Jeong SN, Choi SH (2018). Charlson comorbidity index as a predictor of periodontal disease in elderly participants. J Periodontal Implant Sci.

[8] Song IS, Han K, Ryu JJ, Park JB (2017). Association between underweight and tooth loss among Korean adults. Sci Rep.

[9] do Nascimento TL, Liberalesso NA, Balbinot HJ, Neves HF (2013). Association between underweight and overweight/obesity with oral health among independently living Brazilian elderly. Nutrition.

[10] Kim EK, Lee SK, Jung YS, Lee HK, Song KB, Choi YH (2016). Associations between remaining teeth and salivary flow, activity of daily living, and cognitive impairment among the elderly in a rural area: a pilot study. J Korean Acad Oral Health.

[11] Murray Thomson W (2014). Epidemiology of oral health conditions in older people. Gerodontology.

[12] Emami E, de Souza RF, Kabawat M, Feine JS (2013). The impact of edentulism on oral and general health. Int J Dent.

[13] Anbarserri NM, Ismail KM, Anbarserri H, Alanazi D, AlSaffan AD, Baseer MA (2020). Impact of severity of tooth loss on oralhealth-related quality of life among dental patients. J Family Med Prim Care.

[14] Marcenes W, Kassebaum NJ, Bernabé E, Flaxman A, Naghavi M, Lopez A (2013). Global burden of oral conditions in 1990-2010: a systematic analysis. J Dent Res.

[15] Kassebaum NJ, Bernabé E, Dahiya M, Bhandari B, Murray CJ, Marcenes W (2014). Global burden of severe tooth loss: a systematic review and meta-analysis. J Dent Res.

[16] Kassebaum NJ, Smith AG, Bernabé E, Fleming TD, Reynolds AE, Vos T (2017). Global, regional, and national prevalence, incidence, and disability-adjusted life years for oral conditions for 195 countries, 1990-2015: a systematic analysis for the global burden of diseases, injuries, and risk factors. J Dent Res.

[17] Kim HN, Ha TG, Kim MJ, Jun EJ, Jeong SH, Kim JB (2016). Factors related to number of present teeth in Korean elderly adults aged 55-84 years. Int J Dent Hyg.

[18] Al-Rafee MA (2020). The epidemiology of edentulism and the associated factors: a literature review. J Family Med Prim Care.

[19] Slade GD, Akinkugbe AA, Sanders AE (2014). Projections of U.S. edentulism prevalence following 5 decades of decline. J Dent Res.

[20] Cardoso M, Balducci I, Telles Dde M, Lourenço EJ, Nogueira Júnior L (2016). Edentulism in Brazil: trends, projections and expectations until 2040. Cien Saude Colet.

[21] Yu NH, Shin AR, Ahn SV, Song KB, Choi YH (2021). Estimation and change of edentulism among the Korean population: Korea National Health and Nutrition Examination Survey 2007-2018. Epidemiol Health.

[22] Lee J, Lee JS, Park SH, Shin SA, Kim K (2017). Cohort profile: the National Health Insurance Service-National Sample Cohort (NHIS-NSC), South Korea. Int J Epidemiol.

[23] Song SO, Jung CH, Song YD, Park CY, Kwon HS, Cha BS (2014). Background and data configuration process of a nationwide population-based study using the Korean national health insurance system. Diabetes Metab J.

[24] Koo B, Yoo JJ, Kim M, Lim H, Yoon JH (2020). Analysis of the incidence of dementia in complete edentulous patients using the National Health Insurance Service-Elderly Cohort Database (NHIS-ECD). J Korean Acad Prosthodont.

[25] National Health Insurance Service Guide for health insurance about denture (National Health Insurance Act Enforcement Decree, Article 19 (1) 2) (2012) [cited 2021 Oct 27]. https://www.nhis.or.kr/static/html/wbma/c/wbmac0217_2.pdf.

[26] Keyfitz N (1966). 3. Sampling variance of standardized mortality rates. Hum Biol.

[27] Statistics Korea (2021). 2021 Statistics on the aged.

[28] Kim DJ (2017). New paradigm and policy suggestions for research on senile chronic disease. Survey report.

[29] Yoon JH, Kim Y, Kim DW, Kim MJ (2016). Analysis of the risk of tooth loss in chronic diseases using data from the National Health Insurance Service. Survey report.

[30] Peng J, Song J, Han J, Chen Z, Yin X, Zhu J (2019). The relationship between tooth loss and mortality from all causes, cardiovascular diseases, and coronary heart disease in the general population: systematic review and dose-response meta-analysis of prospective cohort studies. Biosci Rep.

[31] Baelum V, van Palenstein Helderman W, Hugoson A, Yee R, Fejerskov O (2007). A global perspective on changes in the burden of caries and periodontitis: implications for dentistry. J Oral Rehabil.

[32] Choi SS, Sung MA (2020). The effects of chronic diseases experience according to oral condition, self-efficacy scale for self-care and subjective oral health level elderly in some regions. J Korean Oral Health Sci.

[33] Kassebaum NJ, Bernabé E, Dahiya M, Bhandari B, Murray CJ, Marcenes W (2014). Global burden of severe tooth loss: a systematic review and meta-analysis. J Dent Res.

[34] Cooray U, Watt RG, Tsakos G, Heilmann A, Hariyama M, Yamamoto T (2021). Importance of socioeconomic factors in predicting tooth loss among older adults in Japan: evidence from a machine learning analysis. Soc Sci Med.

[35] Kim YH, Han K, Vu D, Cho KH, Lee SH (2018). Number of remaining teeth and its association with socioeconomic status in South Korean adults: data from the Korean National Health and Nutrition Examination Survey 2012-2013. PLoS One.

[36] Wu B, Hybels C, Liang J, Landerman L, Plassman B (2014). Social stratification and tooth loss among middle-aged and older Americans from 1988 to 2004. Community Dent Oral Epidemiol.

[37] Laguzzi PN, Schuch HS, Medina LD, de Amores AR, Demarco FF, Lorenzo S (2016). Tooth loss and associated factors in elders: results from a national survey in Uruguay. J Public Health Dent.

[38] Kim SY, Son SO, Lee HE, Park JE (2019). Agenda-based regional status and welfare indicators study in 2019. Survey report.

[39] Seo HW, Kim YS (2020). Changes in dental care utilization and expenditure by the expansion policy of the health insurance coverage: Korea Health Panel Survey 2012-2016. J Korean Soc Dent Hyg.

